# *In vitro* assessment of pesticides capacity to act as agonists/antagonists of the thyroid hormone nuclear receptors

**DOI:** 10.1016/j.isci.2021.102957

**Published:** 2021-08-08

**Authors:** Yanis Zekri, Laure Dall Agnol, Frédéric Flamant, Romain Guyot

**Affiliations:** 1 Institut de Génomique Fonctionnelle de Lyon, Univ Lyon, CNRS UMR 5242, INRAE USC 1370 École Normale Supérieure de Lyon, Université Claude Bernard Lyon 1, 46 allee d'Italie, 69364 Lyon, France

**Keywords:** biological sciences, toxicology, environmental toxicology

## Abstract

Chemicals acting as thyroid hormone disruptors (THDs) are of a particular concern for public health, considering the importance of this hormone in neurodevelopment and metabolic processes. They might either alter the circulating level of thyroid hormone (TH) or interfere with the cellular response to the hormonal stimulation. In order to assess this later possibility we selected 39 pesticides and combined several *in vitro* tests. Reporter assays respectively addressed the transactivation capacity of the full-length TH nuclear receptor TRα1, the transactivation capacity of its C-terminal ligand binding domain, or the ability of the hormone to destabilize the interaction between TRα1 and the transcriptional corepressor NcoR. Although some pesticides elicit a cellular response, which sometimes interferes with TH signaling, RNA-seq analysis provided no evidence that they can act as TRα1 agonists or antagonists. Their neurodevelopmental toxicity in mammals cannot be explained by an alteration of the response to TH.

## Highlights

• Pesticides were tested for their capacity to interfere with thyroid hormone receptors
• Three reporter assays were combined to identify possible agonists/antagonists
• The tested pesticides are not major disruptors of thyroid hormone signaling

## Graphical abstract

**Figure:**
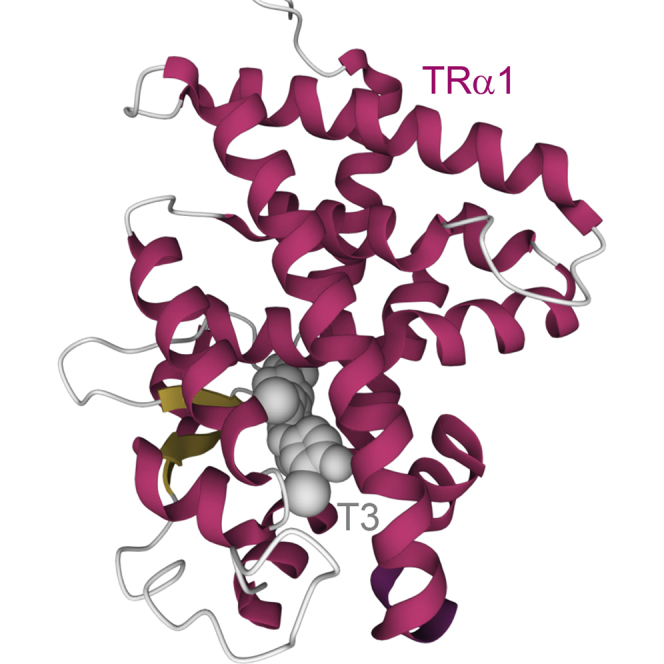

## Introduction

It is now recognized that a number of environmental chemicals act as endocrine disruptors. Among these, thyroid hormone disruptors (THDs) interfere with the thyroid system and have broad adverse effects. In particular, neurodevelopment is highly sensitive to a deficit (Gilbert et al., 2020) or an excess (Laureano-Melo et al., 2019) of thyroid hormone (TH). Therefore, early life exposure to THD might have irreversible consequences on cognitive functions. A number of non-exclusive mechanisms have been proposed to explain the adverse effects of THD, which can be classified in two categories:

Some chemicals interfere with the synthesis or degradation of TH. A number of underlying mechanisms have been documented: xenobiotics can alter the hypothalamus-pituitary-thyroid axis that regulates the thyroid gland activity, inhibit iodine uptake, impair the TH production of the thyroid gland or accelerate the catabolism of TH. The outcome of all these processes is a reduction in the circulating levels of TH or an unbalance between T4 (thyroxine) and T3 (3,3′,5′-Triiodothyronine), the most active form of TH. In exposed rodents, this is often associated to histological alterations of the thyroid gland.

Other chemicals might interfere with the intracellular pathway by which T3 activates the transcription of genes in many cell types. T3 mainly acts in cell nuclei by binding to the C-terminal domain of nuclear receptors (TRs, including TRα1, TRβ1, and TRβ2). Upon T3 binding, the conformation of the C-terminal domain changes (Yen et al., 2006). As a result, chromatin-bound TRs release transcription corepressors, and recruit transcription coactivators, upregulating the transcription of a number of genes (Perissi and Rosenfeld, 2005). Like other endocrine disruptors (Toporova and Balaguer, 2020), THD might compete with T3 to bind the C-terminal domain of TRs and exert an agonist or antagonist influence on T3 signaling (Freitas et al., 2011; Gorini et al., 2020; Guyot et al., 2014; Ibhazehiebo et al., 2010, 2011).

The maintenance of stable levels on TH is dependent on a feedback regulation exerted by TRs, which limits TRH production in the hypothalamus and inhibits TSH secretion by the pituitary gland. The molecular mechanisms by which the receptors exert this negative regulation remain unclear (Ortiga-Carvalho et al., 2016). Therefore, although TR agonists or antagonists would be expected to have major adverse effects on brain development, they will not necessarily modifying the circulating levels of TH. The risk associated to this second class of THD, which only alter the cellular response to T3, is thus better assessed in cultured cells than in rodents, where toxicological tests mainly consider the modes of action from the first class.

Epidemiological associations and toxicological studies indicate that a number of pesticides have a detrimental influence on neurodevelopment. Due to the chemical diversity of pesticides, it is likely that a number of underlying mechanisms exist (Pellizzari et al., 2019; Reigart and Roberts, 2001). Notably, while pesticides have been designed to target specific biochemical pathways in plants or insects, they might lack species specificity and exert a similar toxicity in mammals at high concentration. An alternative hypothesis which has been recently raised, is that they might exert an adverse effect on mammalian neurodevelopment by acting as THD (Leemans et al., 2019). Although TH circulating levels or thyroid gland histology are sometimes altered by pesticides (Moser et al., 2015; Paul et al., 2012; Yaglova and Yaglov, 2017) these effect do not seems to correlate with a direct binding to TRs, which have only been observed at high concentrations for few compounds (Xiang et al., 2017). It is thus tempting to speculate that some pesticides belong to the second class of chemicals, which interfere with the cellular response to T3 without binding to TRs. If this hypothesis is correct, the danger of pesticides exposure would be widely underestimated under the current registration procedures, which mainly relies on *in vivo* testing, and thus favors the detection of the first class of THDs.

We performed here an *in vitro* assessment of the capacity of common pesticides to act as TRα1 agonists or antagonists. We used a combination of three cell assays to limit the risk of false positives. Combined with genome-wide analyses of gene expression, our data indicate that, unlike estrogenic disruptors, THD do not frequently act as agonists or antagonists of the nuclear receptors of TH, but rather exert a partial and indirect influence on T3 response.

## Results

### Transactivation assays

A set of 39 pesticides was selected among pesticides, which are of common or limited use in agriculture (Crepet et al., 2013; Medina-Pastor et al., 2020) and covers the main class of pesticides: carbamates, neonicotinoids, organochlorines, organophosphates, pyrethroids, quinolones, strobilurins, and triazols (Table S1). Two reporter cell lines which were previously characterized (Guyot et al., 2014) were used to test their ability to interfere with T3 signaling. They offer complementary advantages: C17.2α-Hrluc cells are murine cells of neural origin. They host a reporter construct based on the natural promoter of the *Hairless* gene, a well-characterized target gene of TRα1. HEK293–Gal4TRα1luc cells derive from a human fetal kidney cell line. They express a hybrid receptor, which can upregulate a UAS-luciferase reporter, also integrated in the cell genome, after binding of T3 to the C-terminal domain of TRα1. Although this artificial setting does not consider all the molecular events, which can potentially interfere with TRα1-mediated transactivation, it is also more sensitive to T3. We first determined the maximal tolerable concentration for the two reporter cell lines, defined as 10% of the lowest concentration with visible toxicity (Table S1). Two inhibitory TRα1 ligands were used as positive controls (Figure S1): NH-3 prevents the interaction of TRα1 with both coactivators and corepressors (Nguyen et al., 2002) and thus prevents both the T3-induced response and the transcriptional repression exerted by TRα1 in absence of T3. 1-850 is a competitive antagonist (Schapira et al., 2000). Each reporter cell line was used in two modes: either in the absence or presence of T3, to respectively identify TRα1 agonists and antagonists. In each case, negative controls, which were performed with equal amount of solvent, were used as reference. Overall the variability of the measured T3 response was ±10% which allowed us to define thresholds beyond which luciferase activity was considered as significantly altered. A subset of the selected pesticides was active on C17.2α-Hrluc (Table 1) and or HEK293–Gal4TRα1luc cells (Table 2) either in the absence or the presence of T3. In these assays, both 1-850 and NH-3 act as antagonists, reducing luciferase activity in the presence of T3. By contrast, most active pesticides exert a negative influence on luciferase activity in both the absence and presence of T3. This suggests that these are not true TRα1 antagonists but unspecific inhibitors of the reporter systems, which should be considered as false positives.

**Table 1:** Antagonist activity of selected chemicals on C17.2α-Hrluc reporter cells

Molarity of chemical	10-8M		10-7M		10-6M		10-5M	
T3 10-8M	–	+	–	+	–	+	–	+
1-850	1.23 ± 0.04	1.13 ± 0.04	1.15 ± 0.08	1.09 ± 0.02	1.09 ± 0.06	1.14 ± 0.06	**0.87 ± 0.03**	**0.83 ± 0.03**
NH3	1.16 ± 0.03	1.11 ± 0.06	1.22 ± 0.07	1.06 ± 0.03	1.29 ± 0.02	0.90 ± 0.02	0.90 ± 0.05	**0.50 ± 0.06**
Azoxystrobin			1.24 ± 0.13	1.07 ± 0.12	0.99 ± 0.2	1.04 ± 0.26	**0.40 ± 0.02**	0.85 ± 0.07
Benoxacor					0.95 ± 0.15	0.95 ± 0.12	1.09 ± 0.16	1.01 ± 0.08
Beta Endosulfan	1.02 ± 0.04	0.97 ± 0.07	1.16 ± 0.19	0.99 ± 0.10	1.01 ± 0.13	0.79 ± 0.07		
Bifenthrin			0.84 ± 0.04	1.10 ± 0.07	**0.69 ± 0.10**	1.15 ± 0.12	**0.59 ± 0.10**	**0.82 ± 0.10**
Bitertanol	0.89 ± 0.06	0.87 ± 0.09	0.88 ± 0.01	1.20 ± 0.11	0.86 ± 0.04	1.02 ± 0.17	0.87 ± 0.09	1.07 ± 0.02
Captafol			0.89 ± 0.07	0.97 ± 0.17	0.95 ± 0.16	1.00 ± 0.16		
Captan			1.02 ± 0.13	0.93 ± 0.12	1.01 ± 0.06	0.79 ± 0.13		
Chlorothalonil			1.15 ± 0.2	0.93 ± 0.05	**1.44 ± 0.05**	0.84 ± 0.06		
Chlorpyriphos							**0.66 ± 0.21**	**0.63 ± 0.07**
Chlorpyriphos methyl							**0.67 ± 0.15**	**0.64 ± 0.17**
Cypermethrin							0.81 ± 0.07	**0.65 ± 0.13**
Dichlorodiphenyltrichloroethane					1.06 ± 0.05	0.92 ± 0.06		
Deltamethrin							**0.78 ± 0.04**	**0.78 ± 0.21**
Dieldrin	0.83 ± 0.08	1.10 ± 0.22	**0.83 ± 0.03**	0.96 ± 0.17	**0.60 ± 0.11**	**0.71 ± 0.16**		
Dienochlor			1.04 ± 0.13	1.02 ± 0.01	0.95 ± 0.17	0.99 ± 0.06		
Dinoseb					**0.75 ± 0.10**	0.79 ± 0.14		
Disulfoton	0.95 ± 0.07	0.84 ± 0.07	0.97 ± 0.04	0.83 ± 0.07	0.92 ± 0.07	0.77 ± 0.05	0.92 ± 0.13	0.84 ± 0.09
Emamectin benzoate			0.94 ± 0.1	0.83 ± 0.04	**0.79 ± 0.06**	**0.67 ± 0.08**		
Fenitrothion			0.91 ± 0.08	1.06 ± 0.21	0.89 ± 0.10	0.90 ± 0.07	**0.53 ± 0.04**	**0.49 ± 0.06**
Folpet					1.09 ± 0.09	**0.79 ± 0.07**	1.09 ± 0.17	0.98 ± 0.21
Formetanate hydroxide	1.01 ± 0.05	1.01 ± 0.16	1.06 ± 0.07	0.96 ± 0.09	1.13 ± 0.07	0.89 ± 0.08	1.14 ± 0.15	0.87 ± 0.08
Imidacloprid			**0.70 ± 0.12**	0.90 ± 0.33	**0.77 ± 0.06**	0.84 ± 0.12	**0.67 ± 0.09**	**0.72 ± 0.09**
Indoxacarb	1.04 ± 0.15	0.85 ± 0.04	1.12 ± 0.10	0.91 ± 0.10	0.99 ± 0.14	0.92 ± 0.10		
Malathion							**0.73 ± 0.13**	**0.71 ± 0.08**
Penconazole			1.03 ± 0.09	1.04 ± 0.07	0.95 ± 0.10	1.04 ± 0.07	**0.48 ± 0.04**	**0.23 ± 0.01**
Phosalone^a^			1.27 ± 0.10	1.09 ± 0.03	1.17 ± 0.08	1.16 ± 0.03	1.00 ± 0.05	0.55 ± 0.07
Picoxystrobin			1.19 ± 0.11	**0.57 ± 0.24**	0.61 ± 0.1	**0.49 ± 0.01**		
Piperonyl butoxide			**0.69 ± 0.08**	**0.69 ± 0.15**	**0.45 ± 0.05**	**0.57 ± 0.01**	**0.46 ± 0.07**	**0.49 ± 0.03**
Protioconazol	1.15 ± 0.19	1.30 ± 0.13	1.21 ± 0.15	1.18 ± 0.1	1.25 ± 0.12	1.13 ± 0.02		
Pyraclostrobin	0.94 ± 0.03	0.93 ± 0.15	**0.79 ± 0.06**	**0.64 ± 0.1**	**0.53 ± 0.01**	0.88 ± 0.21		
Pyridaben					**0.45 ± 0.01**	**0.45 ± 0.11**		
Quinoxyfen			**0.72 ± 0.05**	**0.68 ± 0.05**	**0.55 ± 0.01**	**0.60 ± 0.02**	**0.44 ± 0.04**	**0.44 ± 0.10**
Tau-fluvalinate							1.09 ± 0.26	**0.76 ± 0.03**
Tris(1,3-dichloro-2-propyl)phosphate	1.26 ± 0.19	1.16 ± 0.04	1.28 ± 0.21	1.14 ± 0.02	1.28 ± 0.16	0.98 ± 0.04		
Thiram					1.29 ± 0.29	1.19 ± 0.06		
Triclosan^a^	1.06 ± 0.12	0.83 ± 0.04	1.00 ± 0.12	0.83 ± 0.02	1.11 ± 0.07	0.83 ± 0.08	0.94 ± 0.07	**0.74 ± 0.06**
Trifloxystrobin					**0.64 ± 0.07**	**0.66 ± 0.04**		
Vinclozolin	0.92 ± 0.14	0.90 ± 0.02	0.81 ± 0.10	0.93 ± 0.05	0.90 ± 0.02	0.87 ± 0.08	0.93 ± 0.05	0.92 ± 0.14
Ziram					1.14 ± 0.26	1.05 ± 0.10		

Data are the relative activity compared to a matched control with equal concentration of the DMSO solvent (0.1%).

a Actual concentration is half of the one indicated. Bold characters indicate deviation from controls, by at least 10%. All concentrations are <10% of the cytotoxic dose.

**Table 2:** Activity of selected chemicals on HEK293–Gal4TRα1luc reporter cells

Molarity of chemical	10-8M		10-7M		10-6M		10-5M	
T3 10-9M	–	+	–	+	–	+	–	+
1-850	0.93 ± 0.06	0.96 ± 0.00	0.91 ± 0.01	1.02 ± 0.05	0.89 ± 0.02	0.97 ± 0.07	**0.70 ± 0.02**	**0.23 ± 0.02**
NH3	0.94 ± 0.06	0.94 ± 0.01	1.03 ± 0.03	0.90 ± 0.03	**1.44 ± 0.2**	**0.53 ± 0.07**		
Azoxystrobin	1.15 ± 0.09	0.80 ± 0.03	1.04 ± 0.16	0.89 ± 0.07	1.12 ± 0.24	1.09 ± 0.12	**0.69 ± 0.02**	**0.51 ± 0.02**
Benoxacor	0.79 ± 0.13	0.92 ± 0.06	0.95 ± 0.09	0.95 ± 0.03	1.07 ± 0.08	0.99 ± 0.03		
Beta Endosulfan	0.97 ± 0.05	1.01 ± 0.05	0.93 ± 0.08	0.90 ± 0.01	0.99 ± 0.04	0.92 ± 0.07		
Bifenthrin							0.96 ± 0.13	1.12 ± 0.02
Bitertanol	0.87 ± 0.03	0.94 ± 0.05	0.87 ± 0.04	0.99 ± 0.05	0.89 ± 0.08	0.97 ± 0.04		
Captafol	**0.63 ± 0.15**	**0.70 ± 0.05**	**0.80 ± 0.1**	**0.66 ± 0.04**	0.98 ± 0.02	0.84 ± 0.14		
Captan	0.86 ± 0.06	**0.84 ± 0.04**	0.90 ± 0.01	**0.70 ± 0.02**	0.96 ± 0.04	0.84 ± 0.07		
Chlorothalonil	**0.71 ± 0.08**	**0.34 ± 0.08**	**0.38 ± 0.06**	**0.33 ± 0.03**	**0.35 ± 0.05**	**0.31 ± 0.06**		
Chlorpyriphos							0.89 ± 0.01	0.96 ± 0.11
Chlorpyriphos methyl							0.95 ± 0.13	0.88 ± 0.02
Cypermethrin							1.01 ± 0.01	0.89 ± 0.13
Dichlorodiphenyltrichloroethane			0.88 ± 0.08	1.11 ± 0.12				
Deltamethrin							**0.80 ± 0.02**	**0.80 ± 0.07**
Dieldrin^a^					1.16 ± 0.04	1.31 ± 0.07	1.05 ± 0.07	1,38 ± 0.10
Dienochlor					0.89 ± 0.01	**0.55 ± 0.07**		
Dinoseb	1.29 ± 0.03	1.05 ± 0.12	**0.76 ± 0.09**	**0.78 ± 0.02**	**0.81 ± 0.08**	**0.87 ± 0.09**		
Disulfoton	**1.60 ± 0.01**	**0.70 ± 0.05**	0.85 ± 0.45	**0.74 ± 0.08**	**0.67 ± 0.07**	**0.63 ± 0.01**	**1.56 ± 0.10**	0.84 ± 0.12
Emamectin benzoate	0.92 ± 0.10	0.89 ± 0.09	0.93 ± 0.07	**0.76 ± 0.10**	0.92 ± 0.09	**0.82 ± 0.05**		
Fenitrothion^a^					1.03 ± 0.06	0.99 ± 0.12	1.04 ± 0.20	1.16 ± 0.17
Folpet	0.87 ± 0.04	0.82 ± 0.05	0.95 ± 0.07	0.83 ± 0.08	0.98 ± 0.04	0.91 ± 0.07		
Formetanate Hydrochloride	0,97 ± 0.04	**1.26 ± 0.05**	0.97 ± 0.02	**1.23 ± 0.03**	1.04 ± 0.09	1.14 ± 0.07		
Imidacloprid							1.01 ± 0.16	1.04 ± 0.02
Indoxacarb	1.02 ± 0.11	0.90 ± 0.09	0.98 ± 0.07	0.90 ± 0.05	1.04 ± 0.02	0.91 ± 0.03		
Malathion							0.96 ± 0.06	0.97 ± 0.01
Penconazole^a^					0.87 ± 0.09	0.99 ± 0.09	1.08 ± 0.16	0.99 ± 0.04
Phosalone^a^					1.04 ± 0.1	1.14 ± 0.07	**0.68 ± 0.10**	**0.65 ± 0.02**
Picoxystrobin	1.02 ± 0.05	1.25 ± 0.29	0.95 ± 0.27	1.09 ± 0.04	**0.81 ± 0.05**	0.94 ± 0.10		
Piperonyl butoxide							**1.27 ± 0.02**	1.04 ± 0.21
Protioconazol	0.93 ± 0.08	1.16 ± 0.06	0.87 ± 0.10	1.15 ± 0.03	0.88 ± 0.04	1.21 ± 0.15		
Pyraclostrobin	0.98 ± 0.11	0.99 ± 0.15	0.98 ± 0.07	0.97 ± 0.14	1.01 ± 0.11	**0.54 ± 0.01**		
Pyridaben	0.90 ± 0.08	0.95 ± 0.01	**0.82 ± 0.04**	**0.76 ± 0.02**	**0.82 ± 0.02**	**0.86 ± 0.05**	0.86 ± 0.06	**0.80 ± 0.01**
Quinoxyfen^a^					0.96 ± 0.09	0.91 ± 0.40	**1.20 ± 0.06**	**0.65 ± 0.05**
Tau-fluvalinate							1.07 ± 0.04	1.11 ± 0.09
Tris(1,3-dichloro-2-propyl)phosphate	**0.70 ± 0.02**	0.80 ± 0.16	**0.67 ± 0.05**	0.95 ± 0.06	**0.68 ± 0.10**	0.97 ± 0.29		
Thiram	0.92 ± 0.06	**0.60 ± 0.21**	0.95 ± 0.10	**0.45 ± 0.06**				
Triclosan	0.98 ± 0.10	1.03 ± 0.04	0.96 ± 0.14	1.07 ± 0.08	1.02 ± 0.06	1.08 ± 0.08	1.03 ± 0.04	1.09 ± 0.10
Trifloxystrobin	1.02 ± 0.12	0.84 ± 0.07	0.83 ± 0.06	1.13 ± 0.07	0.92 ± 0.04	1.15 ± 0.11		
Vinclozolin	1.11 ± 0.11	**0.56 ± 0.02**	1.01 ± 0.02	**0.76 ± 0.06**	1.00 ± 0.24	**0.76 ± 0.02**	0.96 ± 0.07	**0.71 ± 0.02**
Ziram	1.01 ± 0.09	0.82 ± 0.02	**0.49 ± 0.02**	**0.52 ± 0.02**				

Data are the relative activity compared to a matched control, from the same cell batch, with equal concentration of the DMSO solvent (0.1%).

a Actual concentration is half of the one indicated.. Bold characters indicate deviation from controls, by at least 10%. All concentrations are <10% of the cytotoxic dose.

### Two hybrid corepressor interaction

We reasoned that the existence of such false positives would be identified with a third assay in which the addition of antagonists increases, rather than decreases, the luciferase activity. This was achieved in a two hybrid assay, in which cells were transfected to test the interaction between two hybrid proteins as previously described: the Gal4NcoR, which binds DNA at UAS elements, and the VP16TRα1 transactivator. In this setting, the addition of T3 results in the destabilization of the interaction between the two proteins. By contrast to what happens in both previous transactivation assays, T3 produced a reduction of luciferase activity, as expected from previous data (Bochukova et al., 2012; le Maire et al., 2020). Reciprocally, supplementation with the TR antagonist 1-850 increased the luciferase activity in presence of T3 (Figure S1). We ran this assay for the pesticides that were active in the previous assays (Table 3). Few of the tested products behaved as TRα1 antagonists in this assay. However, the behavior of these chemicals did not match the results obtained in other tests. This is illustrated by disulfoton, which increases luciferase activity at high concentration in this assay, as antagonists do. However, disulfoton does not show antagonist activity in the second test (Table 2) in which it rather behaves as an agonist, upregulating the reporter expression in absence of T3. Although several chemicals were active in the two hybrid test, the changes in luciferase activity were in several cases not significantly different when T3 was added to the medium. This suggests that some chemicals, for example deltamethrin and phosalone, interfere with the reporter system without being TRα1 agonists or antagonists. By contrast, pyraclostrobin activity showed some similarity to the one of NH-3, reducing the interaction between TRα1 and NcoR in absence of T3. This suggested that this pesticide could be a TRα1 agonist. However, taken together (Table S2) the reporter tests did not univocally identify TRα1 agonists/antagonists. We thus used RT-qPCR to address more directly the influence of pesticides on C17.2α cells, on the expression of 2 well-characterized TRα1 target genes: *Klf9* and *Hairless.* In this experiment, the reference antagonists 1-850 and NH-3 clearly limited the response to T3 (Table 4). While we expected that mRNA levels would mirror the results of the first test, this was only the case for the 1-850 and NH-3 reference antagonists. For example quinoxyfen had a negative effect on the reporter expression in C17.2α-Hrluc cells (Table 1) but did not modify the *Hr* mRNA level in C17.2α cells. Owing to the possible presence of confounding artifacts inherent to luciferase-based assays, the divergence between the assays results precludes firm conclusions. The discrepancies between the tests rather suggest a complex situation, in which the tested pesticides modify luciferase activity without necessarily binding to the TRα1 ligand binding domain.

**Table 3:** Capacity to displace the NcoR corepressor from the TRα1 ligand binding domain

Molarity of chemical	10-8M		10-7M		10-6M		10-5M	
T3 10-9M	–	+	–	+	–	+	–	+
1-850	1.06 ± 0.08	1.06 ± 0.03	1.09 ± 0.08	0.98 ± 0.05	1.13 ± 0.05	1.13 ± 0.19	**0.84 ± 0.01**	**1.76 ± 0.10**
NH3	1.11 ± 0.05	0.93 ± 0.03	**0.79 ± 0.05**	**0.73 ± 0.03**	**0.54 ± 0.02**	0.79 ± 0.05	**0.66 ± 0.06**	**1.37 ± 0.05**
Beta Endosulfan	1.04 ± 0.04	1.15 ± 0.07	1.11 ± 0.04	1.22 ± 0.26	1.19 ± 0.15	1.20 ± 0.07		
Dienochlor	1.33 ± 0.25	1.22 ± 0.14	0.85 ± 0.07	0.79 ± 0.14				
Disulfoton	0.79 ± 0.15	1.15 ± 0.12	1.07 ± 0.10	1.20 ± 0.11	1.03 ± 0.14	1.11 ± 0.09	1.03 ± 0.01	**1.20 ± 0.02**
Formetanate Hydrochloride	1.05 ± 0.11	0.93 ± 0.02	0.93 ± 0.08	**0.84 ± 0.04**	0.98 ± 0.18	**0.70 ± 0.02**		
Indoxacarb	1.07 ± 0.11	1.17 ± 0.07	1.09 ± 0.08	1.11 ± 0.08	0.90 ± 0.08	0.89 ± 0.05		
Piperonyl butoxide	1.19 ± 0.26	1.39 ± 0.16	1.09 ± 0.36	0.97 ± 0.14	**1.6 ± 0.27**	**0.51 ± 0.11**		
Protioconazol	1.19 ± 0.10	0.57 ± 0.31	**1.32 ± 0.13**	**0.75 ± 0.11**	1.19 ± 0.12	0.93 ± 0.27		
Pyraclostrobin	**0.81 ± 0.04**	**0.77 ± 0.00**	**0.64 ± 0.03**	**0.89 ± 0.27**				
Pyridaben	0.98 ± 0.15	0.96 ± 0.06	**0.71 ± 0.09**	**0.72 ± 0.02**	**0.77 ± 0.17**	**0.77 ± 0.02**	**0.76 ± 0.13**	**0.65 ± 0.03**
Tris(1,3-dichloro-2-propyl)phosphate	0.93 ± 0.08	0.82 ± 0.14	0.84 ± 0.01	0.90 ± 0.06	0.89 ± 0.05	0.96 ± 0.18		
Triclosan	1.28 ± 0.23	1.05 ± 0.08	**1.64 ± 0.28**	**1.46 ± 0.04**	**1.51 ± 0.16**	1.26 ± 0.43		
Vinclozolin	1.00 ± 0.10	1.09 ± 0.20	0.93 ± 0.09	1.00 ± 0.17	1.03 ± 0.12	1.02 ± 0.07	**0.81 ± 0.02**	0.96 ± 0.02

Data are the relative activity compared to a matched control with equal concentration of the DMSO solvent. Bold characters indicate deviation from controls, by at least 10%. All concentrations are <10% of the cytotoxic dose.

**Table 4:** RT-qPCR measurement of *Hairless* and *Klf9* mRNA T3 response

Molarity of chemical		10-9M	10-8M	10-7M	10-6M	10-5M
1-850	*Hairless*					0.45
	*Klf9*					0.57
NH3	*Hairless*				0.58	
	*Klf9*				0.58	
Azoxystrobin	*Hairless*			0.92	0.93	
	*Klf9*		0.61	1.38	1.25	
Dienochlor	*Hairless*		0.66	0.84	0.87	
	*Klf9*		0.97	0.45	0.41	
Fenitrothion	*Hairless*		1.97	1.18	1.10	
	*Klf9*		1.66	1.18	0.94	
Phosalone	*Hairless*			1.66	1.26	
	*Klf9*			1.25	1.20	
Picoxystrobin	*Hairless*		1.69	1.17	2.68	
	*Klf9*		1.33	1.35	1.20	
Piperonyl butoxide	*Hairless*		1.04	0.93	1.10	
	*Klf9*		1.31	1.00	1.07	
Pyraclostrobin	*Hairless*		1.78	2.42	1.26	
	*Klf9*		1.19	1.18	0.96	
Quinoxyfen	*Hairless*			0.99	0.93	
	*Klf9*			0.96	0.89	
Trifloxystrobin	Hairless	0.36	0.82	1.04	1.19	
	Klf9	1.14	1.23	1.16	1.19	

Relative response of mRNA levels compared to control without pesticide (induction rate by T3 in the presence of pesticide/induction rate by T3 in absence of T3). T3 is added at 10^−9^M and chemicals at the indicated molarity.

### Transcriptome analysis

**Figure 1:**
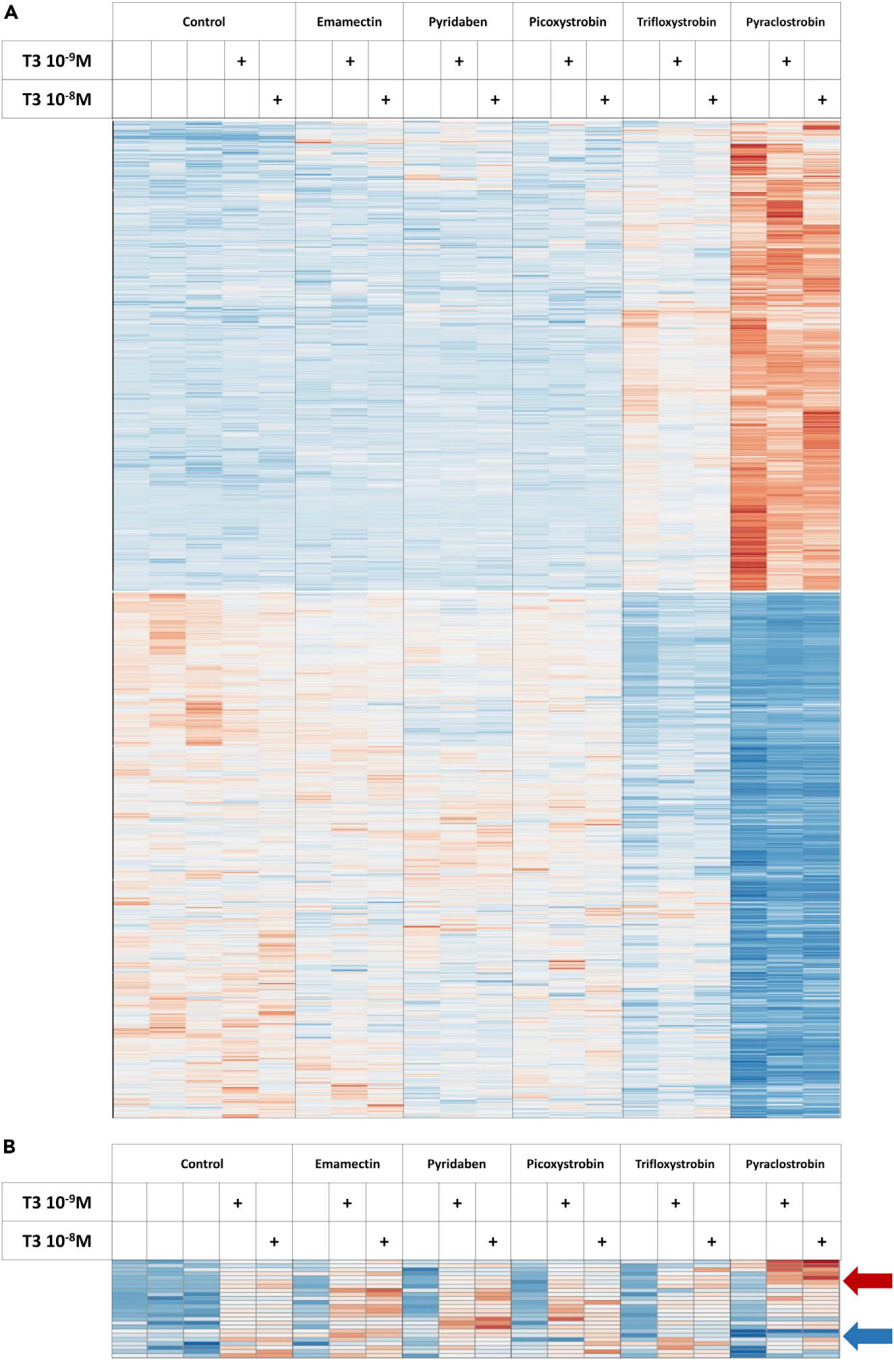
Genome-wide analysis of the pesticides and thyroid hormone response of SHSY5Y human neuroblastoma cells The heatmaps represent the result of a clustering analysis for the response of SHSY5Y human neuroblastoma cells to pesticides and thyroid hormone. Data were normalized and scaled independently for each gene, with the same color code (red: above mean; white: mean; blue: below mean). Ampliseq results obtained from cells exposed to a pesticide (10^−6^M), in absence or in presence of T3 at the indicated concentration. All cDNA libraries were prepared from a single experiment (Run 1 Data S1). Differential expression analysis (Deseq2; First factor pesticide, second factor: T3. Adjusted p value <0.05) identified a number of genes which expression is sensitive to the presence of pesticide (Emamectin: 336 genes; Pyridaben: 225 genes; Picoxystrobin 57 genes; Trifloxystrobin: 5856 genes; Pyraclostrobin: 7290 genes). The reciprocal analysis analysis (Deseq2; First factor T3, second factor: pesticide. Adjusted p value <0.05) identified 25 T3-responsive genes. (A) Pesticide response. Only the 1130 genes displaying at least a 2-fold response to one of the pesticides are included. (B) T3 response. Differential expression analysis (Deseq2; First factor pesticide, second factor: T3. Adjusted p value <0.05) identified a set of 24 genes which were upregulated by T3 in this experiment. All of them have been previously found to be T3-responsive also in C17.2α cells (Chatonnet et al., 2013; Guyot et al., 2014). No systematic bias is observed, which would be expected if one of the pesticide was a TRα1 ligand. Note however that the response to T3 of a subset of genes is sensitive to the presence of pyraclostrobin. A subset of genes becomes more sensitive to T3 stimulation (red arrow), while the T3 response of others is dampened (blue arrow).

To gain a broader and unbiased view of the influence of the complex response of neural cells to the tested chemicals, we selected 7 pesticides which produced the most visible effects in the reporter tests for a genome-wide analysis of gene expression: pyperonyl butoxide, pyridaben, emamectin benzoate, and 3 strobilurins (picoxystrobin, pyraclostrobin, and trifloxystrobin). We first used human neuroblastoma cell line SH-SY5Y, and whole-genome Ampliseq to analyze the response to the pesticides, to T3, and the interference between the two responses. The concentration of pesticides was as high as possible, i.e. 10-fold lower that the lowest cytotoxic concentration. According to differential expression analysis (Deseq2) the compounds except piperonyl butoxide had a significant influence on gene expression (Figure 1A and data not shown). However, there was no indication that TH response was altered as most of the disrupted genes were not responsive to T3. Nevertheless, pyraclostrobin exerts a broad influence on gene expression, which includes an interference with the TH response for some of the T3-responsive genes (Figure 1B).

**Figure 2:**
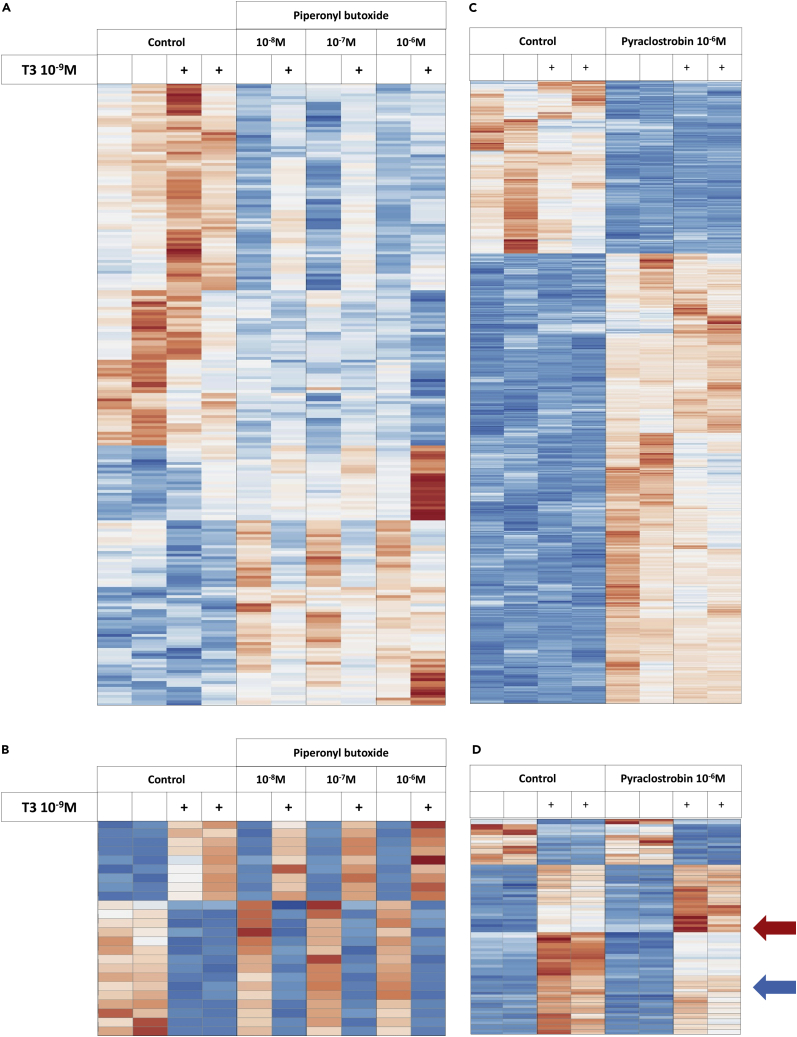
Transcriptome response of C17.2α cells to piperonyl butoxide, pyraclostrobin, and thyroid hormone (A and B) Ampliseq results obtained from cells exposed to piperonyl butoxide at different concentrations, in absence of in presence of T3. Data were normalized and scaled independently for each gene, with the same color code (red: above mean; white: mean; blue: below mean). All cDNA libraries were prepared from a single experiment (Run2, Data S1). Differential expression analysis (Deseq2; First factor pesticide, second factor: T3. Adjusted p value <0.05) identified a number of genes which expression is sensitive to the presence of piperonyl butoxide (10^−8^ 51 genes; 10^−7^ M 75 genes; 10^−6^ M 197 genes)). The reciprocal analysis analysis (Deseq2; First factor T3, second factor: pesticide. Adjusted p value <0.05) identified 25 T3-responsive genes. (A) Pesticide response. (B) T3 response. The clustering analysis does not highlight a systematic bias in T3 response, which would be expected if one of the pesticides was a TRα1 ligand. Note however that the response to piperonyl butoxide is clearly different in present of T3. (C and D) RNA-seq results obtained from cells exposed to Pyraclostrobin (10^−6^ M) in absence of in presence of T3. Only genes with at least a 2 fold-change in expression for one pesticide concentration are plotted (630 out of 4780). Note that the sensitivity to T3 is increased for a subset of genes (red arrow) and decreased for another subset (blue arrow). Data were normalized and scaled independently for each gene on each heatmap, with the same color code (red: above mean; white: mean; blue: below mean).

As SH-SY5Y cells only display moderate TH response, they might not be well suited to identify a minor alteration of this signaling pathway. We thus repeated the experiment for pyraclostrobin and piperonyl butoxide on mouse C17.2α neural cells, as these compounds were active in all reporter tests but with a puzzling pattern. RNA-seq confirmed that these cells, which have been engineered to overexpress TRα1, displayed a robust response to TH (Chatonnet et al., 2013; Guyot et al., 2014). Piperonyl butoxide had a detectable influence on gene expression in these cells. Although T3 had a moderate effect on the response of genes to piperonyl butoxide (Figure 2A) there was no visible reciprocal influence of piperonyl butoxide on the main T3 response genes (Figures 2A and 2B). Pyraclostrobin altered the expression of a larger set of genes (Figure 2C). Although, this pesticide did not systematically alter the cellular response to T3, it potentiated the action of T3 for a subset of T3-responsive genes, and exerted the opposite effect for another subset of these genes (Figure 2D) as already supported by the previous analysis on SH-SY5Y cells. The significance of the influence of the two pesticides on T3 response was addressed using gene set enrichment analysis (GSEA). We used this sensitive non-parametric method to analyze the distribution of the T3-responsive genes within the list of all expressed genes, ranked according to their response to pesticide. The list of genes that are upregulated by T3 in the present experiment was first defined by differential expression analysis. GSEA showed no significant effect of piperonyl butoxide on these genes in absence of T3. On the opposite, it indicated a positive influence of pyraclostrobin on T3-induced genes (p value: 0.02) suggesting a trend toward an agonist-like effect (Figure S2A). As already observed in the clustering analysis, this effect only concerned a subset of the T3-responsive genes.

**Figure 3:**
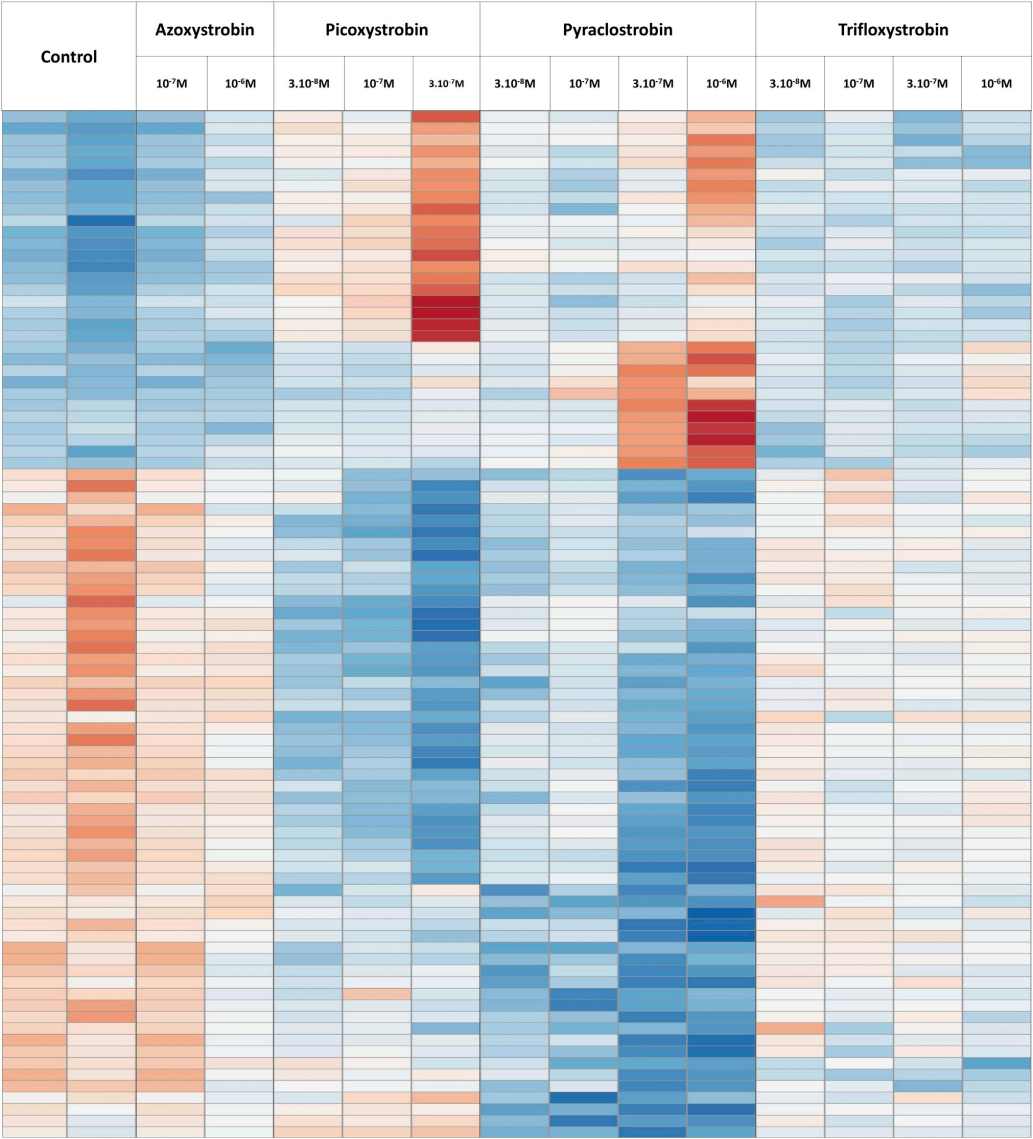
Transcriptome response of C17.2α cells to strobilurins in absence of thyroid hormone Differentially expressed genes were identified by comparing all exposed samples to controls (Deseq2 1 factor, adjusted p value <0.05) after exposing C17.2α to growing concentrations of either azoxystrobin (4 genes), picoxystrobin (3065 genes), pyraclostrobin (962 genes), or trifloxystrobin (272 genes). Only the 90 genes for which a fold-change >2 was measured for at least one pesticide were included in the clustering analysis. Data were normalized and scaled independently for each gene, with the same color code (red: above mean; white: mean; blue: below mean). All cDNA libraries were prepared from a single experiment (Run2, Data S1).

In all the previous experiments, pyraclostrobin stood out as a more active strobilurin. Whether the other strobilurins would exert a similar effect at higher concentrations, or were acting on different pathways, remained unclear. To address this question, we performed another RNA-seq analysis, testing the influence of the four strobilurines at different concentrations in the absence of T3. This analysis showed that the responses to the different strobilurins overlap, suggesting a shared mode of action. Like pyraclostrobin, picoxystrobin was active at low concentration. However, a subset of genes seemed to respond differently to these two compounds (Figure 3).

### Mining published data for putative TH disruptors

**Figure 4:**
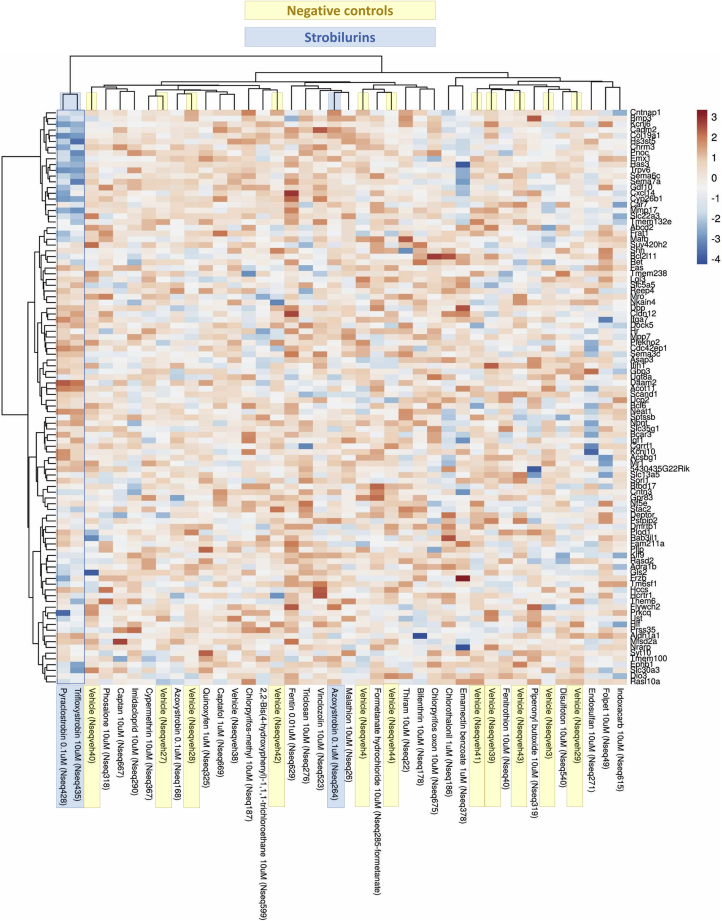
Pesticides activity on cortical neurons A 2D-clustering analysis was performed with the normalized expression data extracted from published results (Pearson et al., 2016), which studied the influence of pesticides on primary cultures of mouse cortical neurons. We extracted the data for the 33 of the 39 pesticides tested in the present study, and 10 negatives controls (yellow boxes). Not that pyraclostrobin and trifloxystrobin are the only strobilurins (blue boxes) to branch out of the controls on the upper dendrogram.

The above data converged to suggest that none of the tested chemicals was a genuine agonist or antagonist of TRα1. However, some molecules, notably pyraclostrobin, interfered with T3 signaling in an indirect manner, modifying the response of a specific subset of TRα1 target genes. The above analysis also showed that GSEA was able to identify such molecules in an unbiased manner, even when the T3 response was only partially altered. We thus took advantage of the availability of a large set of RNA-seq data (GSE70249) which analyzed the influence of 297 chemicals, mainly pesticides at a concentration of 10^−6^M, on primary cell cultures prepared from the cortex of newborn mice (Pearson et al., 2016). Because the culture medium used in these experiments contained T3, present in the B27 supplement and serum (approximatively 10^−9^M), we believe that the assay was mainly suitable to detect TRα1 antagonists, although agonists might potentiate the effect of T3. The response to T3 of this cellular model has been fully characterized by RNA-seq in a different study (Gil-Ibanez et al., 2015), allowing us to define the top 100 T3-induced genes in this system, all of them having a minimal fold-change above 1.5 (pval <0.01, FDR <0.05). The normalized abundance matrix was used to rank 13,304 genes from the most upregulated to the most downregulated in response to each compound (Data S1). We then address the distribution of T3-responsive genes in these ranked lists. This analysis converged to identify some of the compounds that we tested in reporter cells as active on T3 signaling, with antagonist-like properties (emamectin benzoate, piperonyl butoxide, propiconazole and Pyraclostrobin; Table S3). Clustering analysis of the T3-responsive genes for the compounds that we tested in reporter assays showed however that pyraclostrobin and trifloxystrobin are the only chemicals clearly segregating from controls (Figure 4). Again, when considering the full data set, only a small group of chemicals, including pyraclostrobin and trifloxystrobin, branched out of the controls (Figure S4). Based on this GSEA analysis (Figure S2B), we selected 6 compounds, predicted to significantly interfere with TH signaling. Although the different *in vitro* tests provided independent evidence that these compounds were active at different concentrations (Table 5), they did not collectively support the hypothesis that any of these was a genuine agonist or antagonist. For example, vinclozolin, predicted to be an agonist, behaved as an antagonist in test 2. However the effect did not increase with the concentration, and no effect was observed in the other tests. A similar irregular and puzzling response was observed with disulfoton.

**Table 5:** Testing six compounds predicted to be active on T3 signaling by GSEA

Molarity of chemical	10-8M		10-7M		10-6M		10-5M	
T3 10-9M	–	+	–	+	–	+	–	+
Predicted agonists								
Test 1								
Fentin hydroxide	0.94 ± 0.11	**0.78 ± 0.02**	1.01 ± 0.18	**0.68 ± 0.05**				
Formetanate Hydrochloride	1.01 ± 0.05	1.01 ± 0.16	1.06 ± 0.07	0.96 ± 0.09	1.01 ± 0.18	**0.68 ± 0.05**	0.94 ± 0.11	**0.78 ± 0.02**
Vinclozolin	0.92 ± 0.14	0.90 ± 0.02	0.86 ± 0.06	0.93 ± 0.05	0.87 ± 0.09	0.90 ± 0.04	0.99 ± 0.11	0.80 ± 0.14
Test 2								
Fentin hydroxide	**1.86 ± 0.18**	**0.39 ± 0.05**	**2.25 ± 0.14**	**024 ± 0.01**				
Formetanate Hydrochloride	0,97 ± 0.04	**1.26 ± 0.05**	0.97 ± 0.02	**1.23 ± 0.03**	1.04 ± 0.09	1.14 ± 0.07	1.17 ± 0.08	**1.38 ± 0.05**
Vinclozolin	1.11 ± 0.11	**0.56 ± 0.02**	1.01 ± 0.02	**0.76 ± 0.06**	1.00 ± 0.24	**0.76 ± 0.02**	0.96 ± 0.07	**0.71 ± 0.02**
Test 3								
Fentin hydroxide	**0.51 ± 0.07**	**0.52 ± 0.04**	**0.17 ± 0.01**	**0.16 ± 0.01**				
Formetanate Hydrochloride	1.05 ± 0.11	**0.93 ± 0.02**	0.93 ± 0.08	**0.84 ± 0.04**	0.98 ± 0.18	**0.70 ± 0.02**	0.88 ± 11	**0.71 ± 0.10**
Vinclozolin	1.00 ± 0.10	1.09 ± 0.20	0.93 ± 0.09	1.00 ± 0.17	1.03 ± 0.12	1.02 ± 0.07	**0.81 ± 0.02**	0.96 ± 0.02
Predicted antagonists								
Test 1								
Disulfoton	097 ± 0.04	0.83 ± 0.07	0.92 ± 0.07	**0.77 ± 0.05**	0.92 ± 0.13	0.84 ± 0.09	**0.72 ± 0.07**	**0.72 ± 0.05**
Beta Endosulfan	1.02 ± 0.05	0.97 ± 0.07	1.15 ± 0.19	0.99 ± 0.10	1.01 ± 013	**0.78 ± 0.07**		
Indoxacarb	1.04 ± 0.15	**0.85 ± 0.04**	1.12 ± 0.10	0.91 ± 0.10	0.99 ± 0.14	0.92 ± 0.10		
Test 2								
Disulfoton	**1.60 ± 0.01**	**0.70 ± 0.05**	0.85 ± 0.45	**0.74 ± 0.08**	**0.67 ± 0.07**	**0.63 ± 0.01**	**1.56 ± 0.10**	**0.84 ± 0.12**
Beta Endosulfan	0.97 ± 0.05	1.01 ± 0.05	0.90 ± 0.01	0.97 ± 0.05	0.92 ± 0.07	0.93 ± 0.08	0.88 ± 0.09	0.99 ± 0.04
Indoxacarb	1.02 ± 0.11	0.90 ± 0.09	0.98 ± 0.07	0.90 ± 0.05	1.04 ± 0.02	0.91 ± 0.03		
Test 3								
Disulfoton	0.79 ± 0.15	1.15 ± 0.12	1.07 ± 0.10	1.20 ± 0.11	1.03 ± 0.14	1.11 ± 0.09	1.03 ± 0.01	**1.20 ± 0.02**
Beta Endosulfan	1.04 ± 0.04	1.15 ± 0.07	1.11 ± 0.04	1.22 ± 0.26	1.19 ± 0.15	1.20 ± 0.07		
Indoxacarb	1.07 ± 0.11	1.17 ± 0.07	1.09 ± 0.08	1.11 ± 0.08	0.90 ± 0.08	0.89 ± 0.05		

### T3-independent action of strobilurins

**Figure 5:**
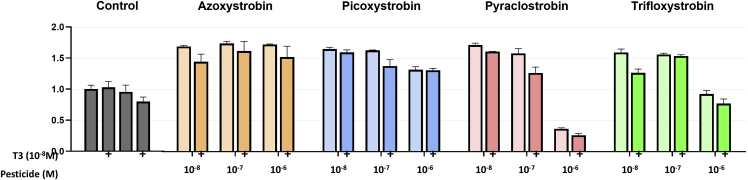
C17.2α cell growth in presence of strobilurins Cells were seeded in 96 wells (5000 cells/well) in medium prepared with hormone-depleted serum and supplemented with the indicated molarity of pesticide and T3. All conditions were triplicated. Cell growth was quantified 4 days later with CellTiter-Glo Luminescent Cell Viability Assay. Data indicate the relative cell density, compared to blanks supplemented with DMSO, at the last of the experiment. While all strobilurins tend to favor cell growth at low concentration, pyraclostrobin and trifloxystrobin have the opposite effect when used at high, non-cytotoxic, concentration. Error bars indicate standard deviations.

The transcriptome analyses that we performed highlighted the capacity of some strobilurins to exert a major influence on gene expression in neural cells, which is unrelated to T3 signaling. Gene Ontology analysis (http://cbl-gorilla.cs.technion.ac.il/) notably indicated that high concentrations of pyraclostrobin downregulated the expression of genes encoding major component of nucleosome assembly, notably histones. As we suspected that this would translate in a modification of cell proliferation, we tested the capacity of strobilurins to influence C17.2α cells growth over 4 days (Figure 5). Interestingly, the four tested strobilurins tended to favor cell growth at low concentration. By contrast high concentrations of pyraclostrobin, and to a lower extend trifloxystrobin, had the opposite effect. These effects were not sensitive to the presence of T3 and observed below the dose of cytotoxicity.

## Discussion

We present here an extensive assessment of the capacity of pesticides to interfere with the cellular response to T3. One main conclusion is that none of the tested compound is behaving as a genuine TRα1 agonist or antagonist. This reinforces the conclusion of the Tox21 screen, performed on 8305 compounds, according to which many environmental chemicals do not act as TRα1 ligand at non-toxic concentrations, either as agonists or antagonists (Paul-Friedman et al., 2019). TRα1 differs from other nuclear receptors, for which a number of environmental ligands have been identified (Liu et al., 2019; Toporova and Balaguer, 2020). This peculiarity probably reflects the small size of the ligand binding pocket of TRs, and the specific chemical properties of their natural iodinated ligands, T4 and T3. In particular, the presence of halogens in the chemical structure (fluoride, iodine, chloride, or bromide), which is frequent in pesticide, is not an indication for a possible binding to TRs.

In line with our previous work data (Guyot et al., 2014), the present study illustrates the benefit provided by transcriptome analysis for *in vitro* toxicology: first it is unbiased, and able to capture any unexpected alteration in the cellular physiology. Second, if performed on relevant cellular models, it helps to prioritize some chemicals for *in vivo* assessment, and limit the use of animals, as recommended by ethical guidelines. Third, as the expression level of each gene of a given signaling pathway can be considered as an independent estimate of the signaling level, it provides outstanding statistical power to detect minor effects on defined pathways. While the tested pesticides have only a marginal influence of TH signaling, our genome-wide analysis shows that many have the potential to compromise neurodevelopment, by exerting a broad influence on gene expression in neural cells, even at low concentration.

In particular, 4 strobilurins, and notably pyraclostrobin, stand out as the most active compounds that we have tested, in agreement with previous conclusions (Pearson et al., 2016). These chemicals were designed to act as fungicides, selectively inhibiting the mitochondrial cytochrome-bc1 complex of fungi. They are however highly toxic to aquatic life. For example 1.5x10^−7^M of pyraclostrobin is lethal in 50% of zebrafishes (Zhang et al., 2017). In mice, a significant reduction in body weight is also observed upon chronic exposure to a food containing more than 10 mg/kg of pyraclostrobin (Bartholomaeus, 2003). Whether these pesticides can reach the fetal brain during pregnancy is currently unknown. According to the Toxcast screen, based on high throughput automated assays, these chemicals are active in a number of *in vitro* tests, affecting many mammalian pathways including TH signaling (Wang et al., 2021). They might also interfere with some nuclear receptors, like PPARs (Luz et al., 2018). Here we found that the neural cells response to pyraclostrobin and trifloxystrobin expands to a fraction of the T3-responsive genes but is mainly T3 independent. Interestingly, we also observed that low concentrations of all the tested strobilurins accelerate the cell proliferation. This property is unexpected for chemicals thought to act mainly on mitochondria and suggest that they can exert an adverse effect in various ways. This class of chemicals typically illustrates the necessity to integrate various approaches, and notably unbiased “omics” to fully appreciate their biological activity, at these cannot be predicted from their structure.

More generally, our study suggests that the definition of the second class of THD, which alter the cellular response to T3, should be reconsidered. Some chemicals do not directly interfere with TRs function but have the capacity to modify the response to T3 for a fraction of the TRs target genes only. If these genes are key mediators of the neurodevelopmental function of T3, this should be a matter of concern.

### Limitations of the study

Our study focused on the search for TRs agonist/antagonist. We did not analyze in details the cause for the discrepancies observed between the three reporter assays for some pesticides, which probably reflect other biological activities, and did not consider the possibility for complex non-monotonic dose responses. In addition, the differential analysis of transcriptome data assumes that the response to pesticides is monotonous, as a single library was prepared for each tested concentration. As the cell culture medium contains serum, which has been depleted of non-polar metabolites, some of the pesticides might bind to serum proteins. This would slightly modify the free concentration of chemicals but not mask their activity. *In vitro* analysis is well suited for mechanistic analysis but insufficient to predict the outcome of *in vivo* toxicity. In particular, it does not take into account the generation of secondary metabolites after catabolism and does not inform on the distribution of xenobiotics in the organisms. Also, as we focused on the interference with TR-mediated cellular response, our study does not inform on the possibility that some of the tested pesticides could act as THDs by interfering with TH synthesis or degradation. Future work is needed to transpose transcriptome analysis to the developing brain.

## STAR★Methods

### Key resources table

**Table:** 

REAGENT or RESOURCE	SOURCE	IDENTIFIER CAS
**Chemicals, peptides, and recombinant proteins**		

Azoxystrobin	Sigma Aldrich	131860-33-8
Benoxacor	Sigma Aldrich	98730-04-2
Beta Endosulfan	Sigma Aldrich	33385-100MG
Bifenthrin	Sigma Aldrich	99267-18-2
Bitertanol	Sigma Aldrich	55179-31-2
Captafol	Sigma Aldrich	01/06/2425
Captan	Sigma Aldrich	133-06-2
Chlorothalonil	Sigma Aldrich	1897-45-6
Chlorpyriphos	Sigma Aldrich	2921-88-2
Chlorpyriphos methyl	Sigma Aldrich	5598-13-0
Cypermethrin	Sigma Aldrich	52315-07-8
Dichlorodiphenyltrichloroethane	Sigma Aldrich	50-29-3
Deltamethrin	Sigma Aldrich	52918-63-5
Dieldrin	Sigma Aldrich	60-57-1
Dienochlor	Sigma Aldrich	2227-17-0
Dinoseb	Sigma Aldrich	88-85-7
Disulfoton	Sigma Aldrich	298-04-4
Emamectin Benzoate	Sigma Aldrich	155569-91-8
Fenitrothion	Sigma Aldrich	122-14-5
Folpet	Sigma Aldrich	133-07-3
Formetanate hydroxide	Sigma Aldrich	23422-53-9
Imidacloprid	Sigma Aldrich	138261-41-3
Indoxacarb	Sigma Aldrich	144171-61-9
Malathion	Sigma Aldrich	121-75-5
Penconazole	Sigma Aldrich	66246-88-6
Phosalone	Sigma Aldrich	2310-17-0
Picoxystrobin	Sigma Aldrich	117428-22-5
Piperonyl Butoxide	Sigma Aldrich	51-03-6
Protioconazol	Sigma Aldrich	178928-70-6
Pyraclostrobin	Sigma Aldrich	175013-18-0
Pyridaben	Sigma Aldrich	96489-71-3
Quinoxyfen	Sigma Aldrich	124495-18-7
Tau-fluvalinate	Sigma Aldrich	102851-06-9
Tris(1,3-dichloro-2-propyl)phosphate	Sigma Aldrich	13674-87-8
Thiram	Sigma Aldrich	137-26-8
Triclosan	Sigma Aldrich	3380-34-5
Trifloxystrobin	Sigma Aldrich	141517-21-7
Vinclozolin	Sigma Aldrich	50471-44-8
Ziram	Sigma Aldrich	137-30-4
Dimethylsulfoxide	Sigma Aldrich	67-68-5
I-850	Sigma Aldrich	251310-57-3
NH-3	Sigma Aldrich	447415-26-1
Tri-iodo-thyronine	Sigma Aldrich	3,3,5-triiodo-L-thyronine

**Critical commercial assays**		

CellTiter-Glo Luminescent Cell Viability Assay	Promega	G7570
Renilla Luciferase Assay System	Promega	E2810
Luciferase Assay System	Promega	E1500
β-Galactosidase Enzyme Assay	Promega	E2000
Ion AmpliSeq™ Transcriptome Human Gene Expression Kit	Thermo Fischer Scientific	A26325
total RNA SENSE kit	Lexogen	001.24

**Deposited data**		

RNA-seq raw data	Gene expression omnibus	GSE171038

**Experimental models: Cell lines**		

C17.2α-Hrluc	Our lab	Guyot et al. (2014)
HEK293–Gal4TRα1luc	Our lab	Guyot et al. (2014)
HEK293	ATCC	CRL-1573™
SHSY5Y	ATCC	CRL-2266™

**Oligonucleotides**		

5’CAGCGTCGTGATTAGCGATG	Eurogentec	HPRT sense
5’ CGAGCAAGTCTTTCAGTCCTGTCC	Eurogentec	HPRT antisense
5’CAGCGTCGTGATTAGCGATG	Eurogentec	Hr sense
5’AGAGGTCCAAGGAGCATCAAGG	Eurogentec	Hr antisense
5’CACGCCTCCGAAAAGAGGCACAA	Eurogentec	Klf9 sense
5’ CTTTTCCCCAGTGTGGGTCCGGTA	Eurogentec	Klf9 antisense

**Recombinant DNA**		

Gal4REx5-βglob-luc-SVNeo	Ballaguer’s lab	Markossian et al. (2018)
pBKGal4NcoR	K Chatterjee’s lab	Gurnell et al. (2000)
pBKVP16TRα1	K Chatterjee’s lab	Gurnell et al. (2000)
pBK-βgal	Our lab	Markossian et al. (2018)

**Software and algorithms**		

Htseq-count	Galaxy Version 0.6.1galaxy3	Anders et al. (2015)
Deseq2	Galaxy Version 2.1.8.3	Love et al. (2014)
GSEA	version 4.1.0 UCSD	Mootha et al. (2003)

### Resource availability

#### Lead contact

Further information and requests for resources should be directed to and will be fulfilled by the lead contact, Frédéric Flamant: Frederic.flamant@ens-lyon.fr.

#### Materials availability

This study did not generate new unique reagents.

### Method details

#### Chemicals, cell culture medium and toxicity assays

All chemical solutions were prepared by dissolving pure compounds (Sigma Aldrich St Louis MI USA) in dimethylsulfoxide (DMSO). Cells were cultivated in GlutaMAX Dulbecco modified Eagle medium (GlutaMAX DMEM, Thermo Fisher Scientific) with 10% (HEK293) or 12% (C17.2 or SHSY5Y) of newborn calf serum (Thermo Fisher Scientific). Endogenous TH were depleted from the serum by stripping with activated charcoal (Sigma Aldrich, St Louis MI USA) to prevent background activation of reporter constructs. The toxicity of each chemicals was tested on each cell line using the CellTiter-Glo Luminescent Cell Viability Assay (Promega, Madison WI, USA). Toxic concentrations (>20% mortality after 24 hours) were used to define the maximum concentration usable in reporter assays, defined as 10% of the lowest toxic concentration.

#### Transactivation in neural cells

The C17.2α-Hrluc reporter cell line was described previously (Guyot et al., 2014). It is derived from murine neural stem cells transfected to overexpress in a stable manner the mouse TRα1 receptor. A reporter construct was also introduced in the genome, in which the gene encoding *Renilla luciferase* is driven by 5426nt of the promoter region of the *Hr/Hairless* gene, which is highly sensitive to T3 transactivation (Thompson, 1996). T3 and/or tested compounds were added in the medium 24 hours after seeding cells in 24 well-plates (10^5^ cells/well). DMSO was used in control cells to keep solvent concentration constant. Cells were lysed after 24h of chemical exposure, and luciferase activity was measured in cell lysates (Renilla luciferase assay system, Promega Madison WI, USA).

#### One-hybrid transactivation assay

The HEK293–Gal4TRα1luc cell line was described previously (Guyot et al., 2014). It is derived from human HEK293 cells and integrates two DNA constructs. One is encoding a hybrid receptor in which the DNA binding domain of the yeast Gal4 transcription factor is fused to the ligand binding domain of mouse TRα1 receptor. The second carries the firefly luciferase reading frame, driven by a Gal4 responsive promoter with 5 UAS elements. HEK293–Gal4TRα1luc cells were seeded in 24 well-plates (10^5^ cells/well). T3 and tested compounds were added in the medium 24h later. DMSO was used in control cells to keep solvent concentration constant. Cells were lysed 48h after seeding and luciferase activity was measured with the firefly luciferase assay system (Promega Madison WI, USA).

#### Two hybrid corepressor interaction

The assay was performed in HEK293 cells transfected for the transient expression of several constructs, as described before (le Maire et al., 2020). The pBKGal4NcoR construct encodes a Gal4NcoR hybrid protein, which normally acts as a transcription repressor on expression vectors driven by the UAS DNA binding elements. The pBKVP16TRα1 construct encodes the VP16 transactivation domain of a trans-acting protein from a herpes simplex virus fused to the ligand-binding domain of mouse TRα1. pGal4REx5-βglob-luc-SVNeo construct is an UAS driven luciferase reporter. In this setting, the interaction between Gal4NcoR and the unliganded VP16TRα1 hybrid protein results in an activation of luciferase expression. Addition of T3 destabilizes the interaction, resulting in a reduction in luciferase activity. The pBK-βgal plasmid was also included, which drives the expression of the E coli lacZ. This enabled to use β-galactosidase activity as an internal standard to correct for any variation in transfection efficiency. HEK293 cells were seeded in 24 well-plates (10^5^ cells/well). Cells were transfected the following day with 100ng of DNA containing a mixture of the 4 plasmids (20ng Gal4REx5-βglob-luc-SVNeo, 30ng pBKGal4NcoR, 30ng pBKVP16TRα1, 20ng pBK-βgal) (Gurnell et al., 2000; Markossian et al., 2018) with the TransIT-Lt1 transfection reagent (Mirus Corporation Madison WI, USA). T3 and tested compounds were added in the medium 4-6h later. DMSO was used in control cells to keep solvent concentration constant. Cells were lysed 24h after chemical exposure to measure luciferase activity (Firefly luciferase assay system; Promega Madison WI, USA) and β-galactosidase activity, using ortho-nitrophényl-β-galactoside (Sigma St Louis MI USA) as substrate.

#### Analysis of reporter assays

All presented data represent triplicates, performed to calculate standard deviations. The maximum concentration was 10% of the lowest cytotoxic concentration on the specific cell type, as described above. Different concentrations of a given chemical were tested in the same experiment, on a single cell batch. Preliminary calibration and repetitions of the tests allowed to define a confidence interval for T3 response of ±10%. All values which are estimated (mean ± standard deviation) to be outside of this confidence interval were called significant and confirmed in an independent experiment. Blanks with DMSO and a positive control with only T3 were included for each novel cell batch and used for recalibration.

#### Cell proliferation assay

C17.2α neural cells were seeded in 96 wells plate (5.10^3^ cells/well) and grown for 4 days with either DMSO or pesticides as above in triplicates. Viable cells were then quantified using the CellTiter-Glo Luminescent Cell Viability Assay (Promega, Madison WI, USA).

#### qRT-PCR analysis

C17.2α neural cells, expressing TRα1 (Chatonnet et al., 2013), were seeded in 6-wells plates (3.10^5^ cells/well). T3 and the tested compounds were added in the medium on the next day. DMSO was used in control cells to keep solvent concentration constant. Cells were lysed 24h after chemical exposure and RNA extracted using a Macherey-Nagel NucleoSpin RNA II kit. RNA concentrations were measured with a Nanodrop spectrophotometer (Thermo Fisher Scientific). Each RNA sample was reverse transcribed using murine leukemia virus reverse transcriptase (Promega) and random DNA hexamer primers. Quantitative PCR was performed according to a standard protocol, using the Biorad iQ SYBRGreen kit and the CFX96 thermocycler (Biorad). Hprt, a housekeeping gene, was used as an internal control. For each pair of primers a standard curve was established and PCR efficiency was controlled to be within usable range (90%–110%) before analysis using the 2^−ΔΔ(Ct)^ method (Livak and Schmittgen, 2001).

#### Transcriptome analysis

RNA were extracted from either human SHSY5Y (ATCC® CRL-2266™) cells or mouse C17.2α (Chatonnet et al., 2013) exposed to chemical and/or T3 (10^-9^M or 10^-8^M). Two methods were used for transcriptome analysis: Ion AmpliSeq™ Transcriptome Gene Expression Kit (Thermo Fischer Scientific) which was run on an Ion Proton™ Sequencer, and RNA-seq. In the latter case, cDNA libraries were prepared using the total RNA SENSE kit (Lexogen, Vienna Austria) and single-end deep sequencing was performed on a NextSeq500 (Illumina) sequencer as described (Guyot et al., 2014; Richard et al., 2020). Although both approaches provide similar informations, Ampliseq differ from RNA-seq as it is targeted on predefined annotated genes and relies on an amplification of exonic sequences. Ampliseq and RNA-seq are also analyzed in a slightly different manner, with different genome annotation, which prevent a simple comparison between the datasets obtained by the two methods. Count tables were prepared using htseq-count (Anders et al., 2015). The count tables for the 3 runs are in Data S1. Statistical analysis was performed for each sequencing run independently. Differential gene expression analysis was performed with DEseq2 (Love et al., 2014) using two factors (pesticide and T3 treatments) and the following thresholds: p-adjusted value < 0.05; average expression > 10 reads per million. DEseq2 full tables are in Data S1. Clustvis was used for clustering analysis, using the Ward unsquared method and Euclidian distances to prepare heatmap representations. Data were normalized and scaled independently for each gene, with the same color code (red: above mean; white: mean; blue: below mean).

#### Gene Set Enrichment Analysis

Gene Set Enrichment Analysis (Mootha et al., 2003; Subramanian et al., 2005) was performed with the GSEA Software (version 4.1.0) of University of California San Diego (https://www.gsea-msigdb.org/gsea/index.jsp) using default parameters. We calculated for each compound an Enrichment Score (ES) indicating the overrepresentation of biological functions associated to genes present in the ranked list of up- or downregulated genes. We retained as significant the compounds inducing a coordinated disruption of thyroid hormone response genes expression with a nominal pval ≤ 0.05.

### Quantification and statistical analysis

For transient expression analyses, triplicates of negative controls were used to define of confidence interval. For each assay, results called significant when the mean estimate (± stdev) did not fall within this confidence interval. For RNAseq analysis, differential analysis of gene expression was performed using Deseq2, assuming a negative binomial distribution.

## Data Availability

• Raw RNA-seq data are available at GSE171038. • Ampliseq raw data are in Data S1. • Any additional information required to reanalyze the data reported in this paper is available from the lead contact upon request.

## Supplemental information

Document S1. Figures S1–S3 and Tables S1–S3

Data S1. Raw counts for Ampliseq and RNAseq RUN Figure 1 count files. Ampliseq analysis of SHSY5Y human neuroblastoma cells exposed to pesticides and thyroid hormone, raw data related to Figure 1. RUN Figure 2 count files. Ampliseq analysis of C17.2α cells to piperonyl butoxide, pyraclostrobin and thyroid hormone, raw data related to Figure 2. RUN Figure 3 count files. Ampliseq analysis of C17C17.2α cells exposed to strobilurins in absence of thyroid hormone, raw data related to Figure 3. Differentially expressed genes and normalized counts Figure 1. Complete results of Deseq2 analysis of data from Figure 1. Differentially expressed genes and normalized counts Figure 2. Complete results of Deseq2 analysis of data from Figure 2. Differentially expressed genes and normalized counts Figure 3. Complete results of Deseq2 analysis of data from Figure 2.
