# *In vitro* assessment of pesticides capacity to act as agonists/antagonists of the thyroid hormone nuclear receptors

**DOI:** 10.1016/j.isci.2021.103246

**Published:** 2021-10-13

**Authors:** Yanis Zekri, Laure Dall Agnol, Frédéric Flamant, Romain Guyot

## Main text

(iScience, *24*, 102957-1–102957-20; September 24, 2021)

In the original version of the article, the name of the last author, Romain Guyot, was accidentally placed on the first affiliation line. The authors apologize for any confusion it may have caused. This has since been corrected online.

