# Supplementary material for: In vitro assessment of pesticides capacity to act as agonists/antagonists of the thyroid hormone nuclear receptors
Source: iScience. 2021 Aug 8;24(9):102957. doi: 10.1016/j.isci.2021.102957 (PMC8403745; doi:10.1016/j.isci.2021.102957)
Supplement: Document S1. Figures S1–S3 and Tables S1–S3 [file mmc1.pdf]

iScience, Volume *24*

Supplemental information

In vitro assessment of pesticides capacity to act as agonists/antagonists of the thyroid hormone nuclear receptors

Yanis Zekri, Laure Dall Agnol, Frédéric Flamant, and Romain Guyot

Supplementary data

Supplementary figures

Figure S1 related to Table 1,2 and 3: Validation of reporter tests with reference compounds. T3 increases the luciferase activity of C17.2α-Hrluc cells (upper panel) and HEK293–Gal4TRα1luc cell line (middle panel). 1-850 and NH-3 are TRα1 synthetic ligands which inhibit T3 mediated transactivation in a concentration dependent manner. Lower panel: In HEK293 cells co-transfected with pBKGal4NcoR/pBKVP16TRα1/pGal4REx5-βglob-luc-SVNeo the interaction between the NcoR corepressor domain and the TRα1 ligand binding domain in absence of T3 results in a transactivation of the UAS driven promoter and a high luciferase activity. Addition of T3 (10^{-9}M) destabilizes the interaction between the two hybrid proteins and reduces luciferase activity. 1-850 has little effect in absence of T3, and prevents T3 response in a dose dependent manner. By contrast NH-3 *per se* destabilizes the interaction between NcoR and TRα1 in absence of T3. In presence of T3, it cooperates with T3 to reduce luciferase activity, and has no visible effect. The minor effect of 1-850 and NH-3 in absence of added T3 suggests the persistence of a small amount of T3 in the stripped serum.

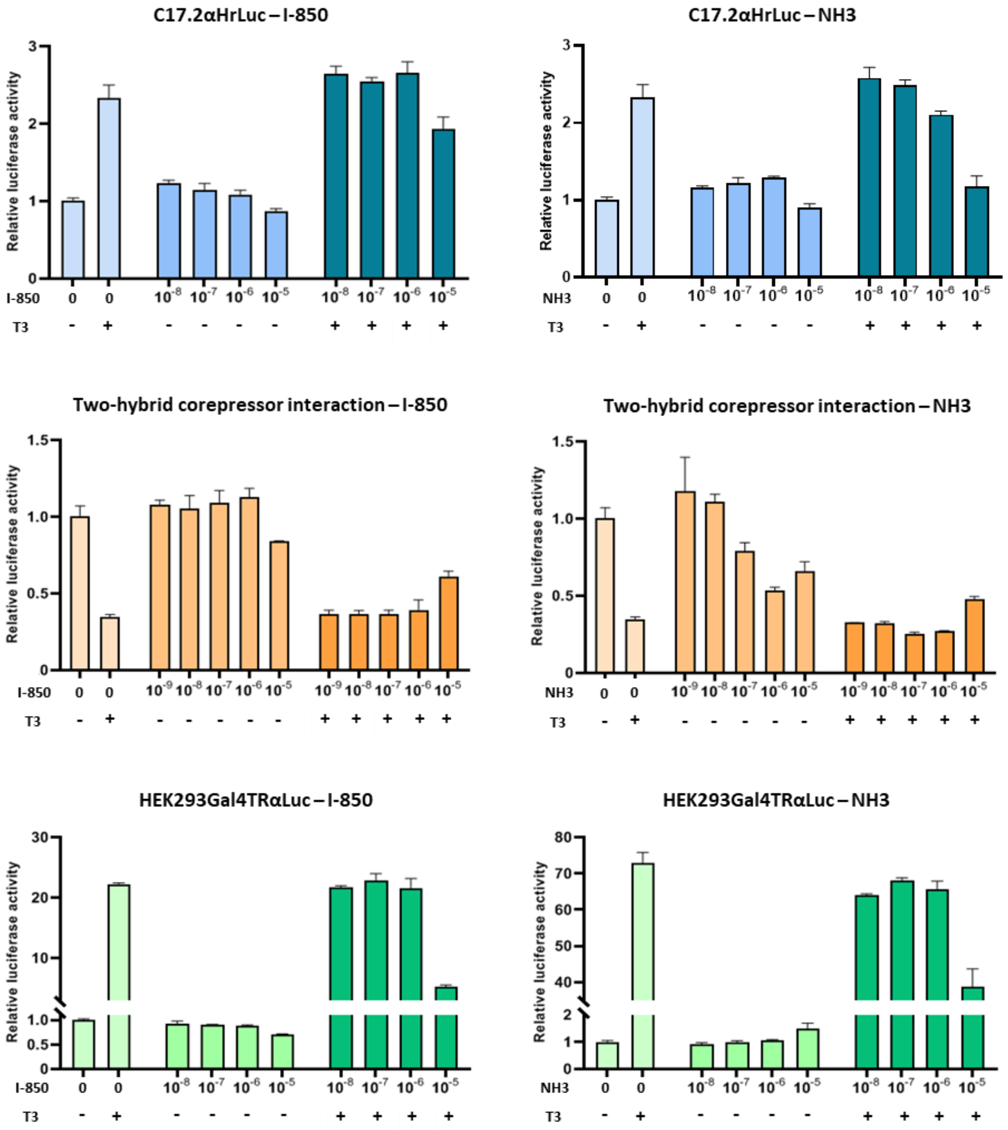

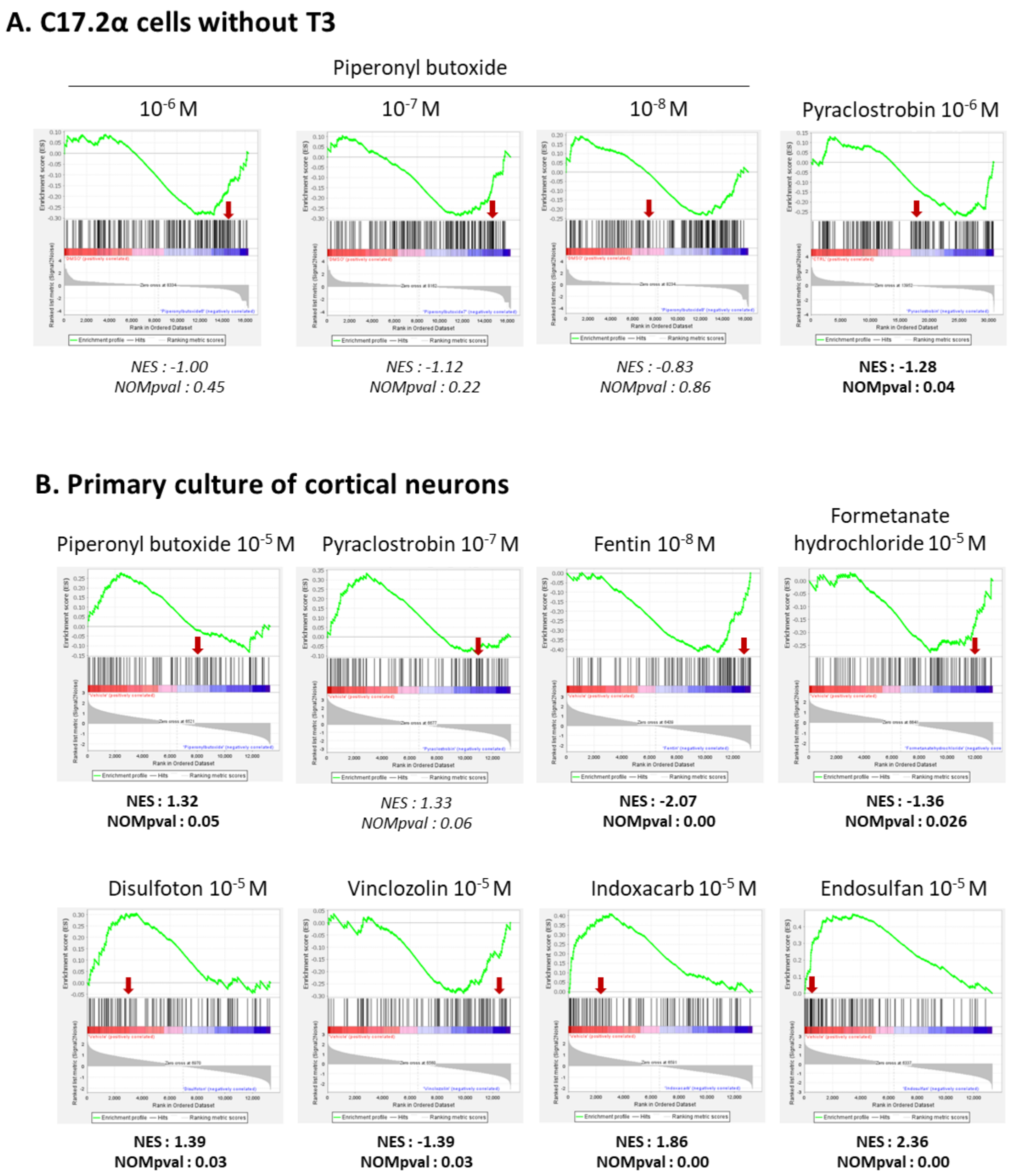

Figure S2 related to figure 2.: Geneset enrichment analysis. Influence of chemicals on T3 responsive genes

(A) C17.2α (B) primary cultures of cortical neurons. Expressed genes are ranked in the bottom gradient from the most upregulated (left) to the most down-regulated (right) in vehicle treated cells compared to chemical exposed cells. Vertical black bars represent the position of each T3 responsive genes in the list of ranked genes: vertical black bars on the left of the gradient are T3 responsive genes more expressed in vehicle treated cells while genes more expressed in chemical exposed cells are on the right. The green curve recapitulates the distribution of T3 responsive genes among the sequenced genes: its peak is either on the left or the right of the gradient, depending of an antagonist or agonist effect of the chemical on T3 responsive gene expression, respectively. The distribution is significantly shifted when adjusted p-value is inferior to 0.05. Adjusted p-values (bold when significant, italics when non-significant) and normalized enrichment score (NES, negative when agonist effect, positive when antagonist effect) are reported within each graph. The red arrow indicates the position of *Hairless*, a gene which expression is highly sensitive to T3.

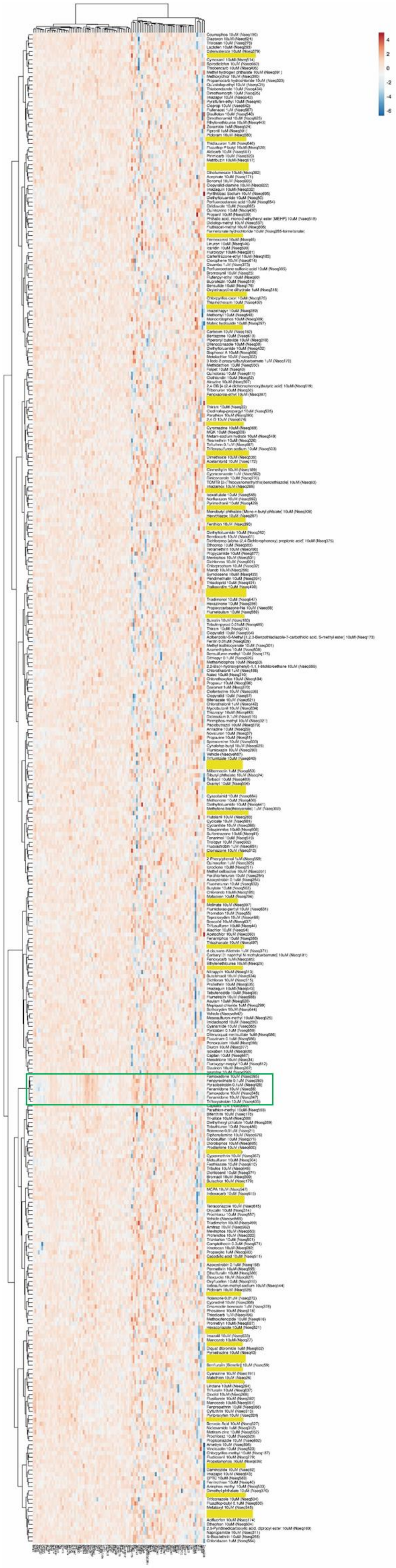

Figure S3 related to figure 4: 2D Clustering of the RNA-seq data of T3 responsive genes for all pesticides tested on primary cultures of cortical neurons. The yellow boxes correspond to negative controls. The green frame highlights a cluster of 5 pesticides which alter the expression of a small group of T3 responsive gene. This cluster contains an acaricide (fenpyroximate) and 4 fungicides (Pyraclostrobin, trifloxystrobin, famoxadone and fenamidone) which all inhibit the mitochondria electron transport chain of their target organisms.

Table S1 Tested chemicals cytotoxicity related to table 1

Compound	Class	Cytotoxic concentration
Azoxystrobin	Strobilurin	10^{-5} M
Benoxacor	Organochlorine	10^{-5} M
Beta Endosulfan	Organochlorine	10^{-4} M
Bifenthrin	Pyrethroid	10^{-5} M
Bitertanol	Other	10^{-6} M
Captafol	Organochlorine	10^{-4} M
Captan	Organochlorine	10^{-4} M
Chlorothalonil	Organochlorine	10^{-6} M
Chlorpyriphos	Organophosphate	10^{-6} M
Chlorpyriphos methyl	Organophosphate	10^{-6} M
Cypermethrin	Pyrethroid	10^{-4} M
Dichlorodiphenyltrichloroethane	Organochlorine	10^{-4} M
Deltamethrin	Pyrethroid	10^{-5} M
Dieldrin	Organochlorine	10^{-6} M
Dienochlor	Organochlorine	10^{-5} M
Dinoseb	Other	10^{-5} M
Disulfoton	Organophosphate	10^{-6} M
Emamectin Benzoate	Other	10^{-5} M
Fenitrothion	Organophosphate	10^{-5} M
Folpet	Organochlorine	10^{-6} M
Formetanate hydroxide	Carbamate	10^{-4} M
Imidacloprid	neonicotinoids	10^{-5} M
Indoxacarb	oxadiazine	10^{-5} M
Malathion	Organophosphate	10^{-4} M
Penconazole	Organochlorine	10^{-6} M
Phosalone	Organophosphate	10^{-4} M
Picoxystrobin	Strobilurin	10^{-5} M
Piperonyl Butoxide	Pyrethroid	10^{-4} M
Protioconazol	Organochlorine	10^{-5} M
Pyraclostrobin	Strobilurin	10^{-5} M
Pyridaben	Organochlorine	10^{-6} M
Quinoxyfen	Organochlorine	10^{-5} M
Tau-fluvalinate	Pyrethroid	10^{-5} M
Tris(1,3-dichloro-2-	Organophosphate	10^{-5} M
Thiram	Other	10^{-5} M
Triclosan	Organochlorine	10^{-6} M
Trifloxystrobin	Strobilurin	10^{-5} M
Vinclozolin	Organochlorine	10^{-5} M
Ziram	Other	10^{-5} M

TableS2: Synoptic view of reporter assays results related to table 1,2 and 3

	C17.2α-Hrluc			HEK293–Gal4TRα1luc			HEK293–Gal4NcoR/VP16TRα1		
Chemical molarity	10-7M	10-6M	10-5M	10-7M	10-6M	10-5M	10-7M	10-6M	10-5M
1-850									
NH3									
**Azoxystrobin**									
Benoxacor									
Beta Endosulfan									
Bifenthrin									
Bitertanol									
Captafol									
Captan									
Chlorothalonil									
Chlorpyriphos									
Chlorpyriphos methyl									
Cypermethrin									
Dichlorodiphenyltrichloroethane									
Deltamethrin									
Dieldrin									
Dienochlor									
Dinoseb									
Disulfoton									
**Emamectin Benzoate**									
Fenithrotion									
Folpet									
Formetanate hydroxide									
Imidacloprid									
Indoxacarb									
Malathion									
Penconazol									
Phosalone									
**Picoxystrobin**									
**Piperonyl Butoxide**									
Protioconazol									
**Pyraclostrobin**									
**Pyridaben**									
Quinoxyfen									
Tau-fluvalinate									
Tris(1,3-dichloro-2-propyl)phosphate									
Thiram									
Triclosan									
Trifloxystrobin									
Vinclozolin									
Ziram									

Bold characters are for chemicals which were selected for transcriptome analysis. White: not tested. Light grey: no significant effect. Dark grey: significant effect, similar in presence or absence of T3. Black: significant effect, different in the presence or absence of T3. NB: Vinclozolin behave as an antagonist in HEK293–Gal4TRα1luc cells and an agonist in HEK293–Gal4NcoR/VP16TRα1/UASluc cells

Table S3 related to figure 5. GSEA analysis of selected pesticides properties on primary cultures of cortical neurons.

	Rank of genes up-regulated by T3				Expected activity
Compound	Enrichment Score	Normalized	Nom. p-value	rank at max	
Azoxystrobin	0.21	0.95	0.54	2848	
Bifenthrin	-0.23	-1.14	0.20	1746	
Captafol	-0.22	-1.08	0.29	3185	
Captan	0.19	0.91	0.65	426	
Chlorothalonil	0.27	1.21	0.12	3035	
Chlorpyrifos	-0.23	-1.12	0.22	3226	
Cypermethrin	-0.22	-1.11	0.23	3430	
Emamectin benzoate	0.32	1.37	**0.05**	1793	antagonist
Fenitrothion	0.21	0.95	0.55	2424	
Folpet	0.23	1.08	0.28	2294	
Imidacloprid	0.24	1.16	0.17	2398	
Malathion	-0.17	-0.86	0.77	3582	
Phosalone	0.23	1.08	0.29	2636	
Piperonyl butoxide	0.28	1.32	**0.05**	2427	antagonist
Propiconazole	0.32	1.54	**0.01**	2353	antagonist
Pyraclostrobin	0.33	1.34	**0.05**	2868	antagonist
Pyridaben	0.25	0.94	0.58	2356	
Quinoxyfen	-0.23	-1.14	0.21	4264	
Thiram	-0.21	-0.90	0.69	2457	
Triclosan	-0.23	-1.15	0.18	2259	
Trifloxystrobin	0.32	1.29	0.08	3125	
Disulfoton	0.31	1.39	**0.04**	3571	antagonist
Endosulfan	0.48	2.36	**0.00**	3434	antagonist
Fentin	-0.42	-2.07	**0.00**	2387	agonist
Formetanate hydrochloride	-0.28	-1.36	**0.03**	4507	agonist
Indoxacarb	0.41	1.86	**0.00**	2978	antagonist
Vinclozolin	-0.29	-1.39	**0.03**	3395	agonist
